# Cochrane corner: video calls for reducing social isolation and loneliness in older people

**DOI:** 10.11604/pamj.supp.2020.35.24283

**Published:** 2020-06-16

**Authors:** Thobile Malinga, Bey-Marrié Schmidt, Charles Shey Wiysonge

**Affiliations:** 1 Cochrane South Africa, South African Medical Research Council, Cape Town, South Africa; 2 School of Public Health and Family Medicine, University of Cape Town, Cape Town, South Africa; 3 Department of Global Health, Stellenbosch University, Cape Town, South Africa

**Keywords:** social isolation, loneliness, pandemic

## Abstract

The occurrence of social isolation and loneliness has increased with the implementation of social distancing measures in multiple countries to contain the COVID-19 pandemic. Social isolation and loneliness are associated with considerable morbidity and mortality. Numerous interventions addressing social isolation and loneliness have been studied, including social facilitation using video calls. A Cochrane review, published in May 2020, assessed the effectiveness of video calls for preventing social isolation and loneliness in the elderly. The authors conducted a comprehensive electronic search and found three eligible studies, none of which was conducted during the COVID-19 pandemic. The review provides limited and uncertain evidence regarding the effectiveness of video call interventions to reduce loneliness in the elderly. Given the need for social distancing among the elderly, even when social distancing measures are lifted in African countries, there is a need for further research on the effectiveness of video calls in reducing social isolation and loneliness among older people.

## Commentary

The rapid spread of the novel coronavirus that emerged in late 2019, and the resulting coronavirus disease 2019 (COVID-19), has been labeled a Public Health Emergency of International Concern (PHEIC) by the World Health Organization (WHO). And as global public health agencies like WHO struggle to contain the outbreak of coronavirus, social distancing is repeatedly suggested as one of the most preventive strategies especially for the elderly [1]. Ironically, however, social isolation may have a negative impact on their health [2], thus numerous interventions addressing loneliness and social isolation have been studied including social facilitation using technology like video calls. In this commentary we highlight the findings of a Cochrane rapid review by Noone and colleagues, which assessed the effectiveness of video calls for reducing social isolation and loneliness in older people [3].

In the review, the authors discussed early findings from three studies that compared the use of video call interventions to usual practice. None of which was conducted during the COVID-19 pandemic. Overall, the studies looked at loneliness, social isolation, and quality of life. Reviewers used a range of terms to categorize the characteristics of the interventions such as video calls through computers, smartphones or tablets [3]. Despite the included studies evaluating interventions aiming to reduce isolation and loneliness, only one out of three studies explicitly targeted people in this situation. The other studies addressed symptoms of depression and there were no studies found on social isolation. The review yielded very low certainty evidence on the effectiveness of video call to reduce loneliness. Despite these findings, fewer studies have shown that using the internet for communication is associated with general improved mental well-being and ability to deal with various situations; in this instance, social isolation [4]. Another study demonstrated that participants reported a significantly greater reduction in overall loneliness, in comparison with the control group, while no difference was observed on the sub-scales of social and emotional isolation [5]. Additionally, a study by Tsai and colleagues demonstrated that the use of smartphone-based videoconferencing intervention reduced feelings of loneliness and improved variables of quality of life [6].

## Conclusion

Given the very low certainty of the evidence, it is difficult to draw conclusions about which intervention can be effective to reduce social isolation and loneliness in the elderly [1]. And research has shown that there is no one-size-fits-all approach to addressing loneliness or social isolation; hence the need to tailor interventions to suit the needs of individuals, specific groups, or the degree of loneliness experienced [7]. Interventions targeting loneliness and social isolation show promise, but more research is needed to provide firm guidance as to which interventions are effective for which populations.

## Competing interests

The authors declare no competing interests.
